# Nanopore Sequencing as a Rapidly Deployable Ebola Outbreak Tool

**DOI:** 10.3201/eid2202.151796

**Published:** 2016-02

**Authors:** Thomas Hoenen, Allison Groseth, Kyle Rosenke, Robert J. Fischer, Andreas Hoenen, Seth D. Judson, Cynthia Martellaro, Darryl Falzarano, Andrea Marzi, R. Burke Squires, Kurt R. Wollenberg, Emmie de Wit, Joseph Prescott, David Safronetz, Neeltje van Doremalen, Trenton Bushmaker, Friederike Feldmann, Kristin McNally, Fatorma K. Bolay, Barry Fields, Tara Sealy, Mark Rayfield, Stuart T. Nichol, Kathryn C. Zoon, Moses Massaquoi, Vincent J. Munster, Heinz Feldmann

**Affiliations:** Friedrich-Loeffler-Institut, Greifswald–Insel Riems, Germany (T. Hoenen, A. Groseth);; National Institutes of Health, Hamilton, Montana, USA (T. Hoenen, A. Groseth, K. Rosenke, R.J. Fischer, S.D. Judson, C. Martellaro, D. Falzarano, A. Marzi, E. de Wit, J. Prescott, D. Safronetz, N. van Doremalen, T. Bushmaker, F. Feldmann, K. McNally, V.J. Munster, H. Feldmann);; Independent scholar, Aachen, Germany (A. Hoenen); University of Saskatchewan, Saskatoon, Saskatchewan, Canada (D. Falzarano);; National Institutes of Health, Bethesda, Maryland, USA (R.B. Squires, K.R. Wollenberg, K.C. Zoon);; The Liberian Institute for Biomedical Research, Charles Ville, Republic of Liberia (F.K. Bolay);; Centers for Disease Control and Prevention, Atlanta, Georgia, USA (B. Fields, T. Sealy, M. Rayfield, S.T. Nichol);; Ministry of Health and Social Welfare, Monrovia, Republic of Liberia (M. Massaquoi)

**Keywords:** Ebola hemorrhagic fever, Ebola virus, Ebolavirus, viruses, high-throughput nucleotide sequencing, nanopore sequencing, molecular diagnostic techniques, DNA, disease outbreaks, Ebola virus disease, Liberia, West Africa

## Abstract

Rapid sequencing of RNA/DNA from pathogen samples obtained during disease outbreaks provides critical scientific and public health information. However, challenges exist for exporting samples to laboratories or establishing conventional sequencers in remote outbreak regions. We successfully used a novel, pocket-sized nanopore sequencer at a field diagnostic laboratory in Liberia during the current Ebola virus outbreak.

Disease outbreaks in resource-limited or remote areas pose unique challenges to outbreak responses. These challenges are exemplified by the ongoing Ebola virus (EBOV) outbreak in West Africa that began in 2014 (*1*) and is unprecedented in its size and duration. Correspondingly, the magnitude of the international response, encompassing ≈50 Ebola treatment units (ETUs) and >2 dozen diagnostic laboratories, has been equally unprecedented. These laboratories often are operated under improvised field conditions to keep them close to active, sometimes remote transmission sites (*2*,*3*).

Rapidly obtaining genome sequences during disease outbreaks is crucial for clarifying patterns of virus evolution, monitoring the validity of diagnostic assays, and investigating transmission chains (*4*,*5*). Further, rapid results may help determine the efficacy of sequence-dependent countermeasures, such as siRNAs or antibody treatments. In the past, obtaining timely genome sequences has been difficult because of political and logistical obstacles that limited the export of samples to laboratories capable of performing these analyses. As an example, during the first year of the outbreak in West Africa, only 2 reports of genome sequences from patients were published (*1*,*6*). Similarly, establishing conventional Sanger or next-generation sequencing technologies in affected countries is logistically challenging because of the size and weight (≈40 to ≈100 kg) of the necessary equipment, the high potential for transport damage related to the sensitive optics many of these machines incorporate, limitations on supportive infrastructure, and complex sample processing procedures. An additional challenge is the required installation or calibration of sequencing machines, which often has to be done by field engineers employed by the manufacturers, who may be reluctant to send their employees into outbreak areas. However, Kugelman et al. recently reported the successful deployment of an Illumina MiSeq, a well-established, conventional next-generation sequencing platform (Illumina, San Diego, CA, USA), to West Africa; the platform became operational in February 2015 (*5*).

Seeking a platform that would be more rapidly deployable and reliable under field conditions, we established protocols and evaluated the feasibility of nanopore sequencing technology under outbreak conditions using a pocket-sized (≈10 × 4 × 2 cm, 75 g) MinION sequencing device (Oxford Nanopore Technologies, [https://www.nanoporetech.com/]). Because of its small size, this device can easily be transported into remote locations; furthermore, it requires no special setup or calibration procedures and can be operational immediately after arrival in an outbreak area. Further, data turnaround is very rapid, and consequently, nanopore sequencing is being developed as a rapid diagnostic tool for management of outbreaks of various diseases (*7*,*8*). The MinION device senses individual DNA molecules based on modulation of ion currents across nanopores as the molecules are passing through. These modulations are dependent on the physical properties of the nucleotides and allow determination of the nucleotide sequence (*9*).

## The Study

**Figure 1:**
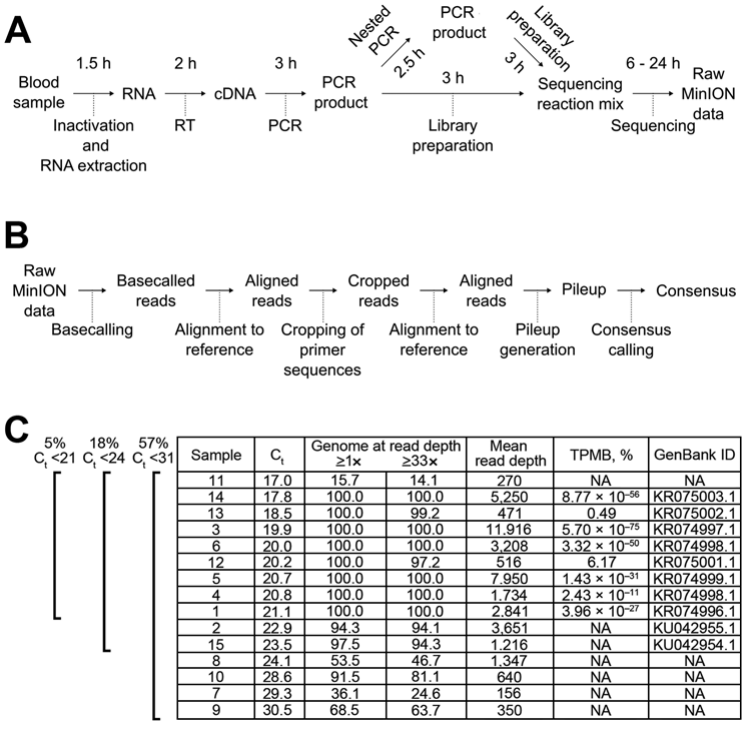
MinION sequencing. A) Experimental and B) bioinformatics workflows. Times indicated are the approximate duration for each procedure. RT, reverse transcription. C) Sequencing results showing Ebola virus load (expressed as C_t_ value), percentage of the genome with a minimum read depth of >1 or >33, mean read depth, theoretical probability for a miscalled base (TPMB), and GenBank accession numbers of complete and nearly complete genomes. Brackets at left indicate percentage of Ebola virus-positive patient samples below each of the 3 cutoff cycle threshold (C_t_) values used in this study (C_t_ <21, <24, <31). Sample 8 was from an oral swab; all others were from blood. NA, not available.

To facilitate sequencing of the RNA genome of EBOV, we developed and tested an approach based on reverse transcription PCR, in which whole virus genomes were amplified in overlapping fragments (Figure 1, panels A, B; Technical Appendix). This approach was first validated in a regular laboratory setting in the Rocky Mountain Laboratories of the National Institutes of Health (NIH) by using blood samples from nonhuman primates experimentally inoculated with EBOV strain Makona-Gueckedou-C07 (*10*,*11*). This validation showed that sequencing information was obtainable for the complete genome with an average of 7,038 reads at every nucleotide position (read depth; Technical Appendix Figure 1, panel A). We observed no sequence differences when comparing the consensus sequence derived from these data to those obtained by using Sanger sequencing (Technical Appendix Figure 1, panel B). Furthermore, by analyzing linearized plasmid DNA of known sequence, we established the accuracy of the MinION device as ≈84% for a single read (Technical Appendix Figure 1, panels C, D). On the basis of this information, and the fact that read depth can compensate for miscalled nucleotides in individual reads by piling up reads covering the same region, we determined the theoretical probability for >1 miscalled base (TPMB) in a complete MinION-sequenced EBOV genome to be <5% when the read depth is >33 at all positions (Technical Appendix Figure 1, panels E and F).

After having validated this approach, MinION devices were taken to the Centers for Disease Control and Prevention (CDC)/NIH field laboratory that provided diagnostic support for ETUs in Monrovia, Liberia, during August 2014–May 2015. All equipment and reagents necessary for sequencing could be easily transported as checked luggage by a single person on a commercial carrier. In Liberia, temperatures in the laboratory area used for sequencing ranged from 28 to 32°C, necessitating the use of an improvised heat sink for the devices, which consisted of a metal plate of ≈30 × 30 cm (Technical Appendix Figure 2, panels A, B). Under field conditions, we initially failed to produce complete genomes with high confidence because of problems with PCR yields (online Technical Appendix Figure 3, panels A, B). However, by implementing a second PCR step, we circumvented this problem and obtained high quality complete genome sequences for 8 of 9 high-virus load samples (cycle threshold <21) (Figure 1, panel C; Technical Appendix Figure 4, panel A). In lower virus load samples, we could obtain only incomplete genome sequences; however, even in those samples regions for which sequencing information was available generally showed high read depths (Technical Appendix Figure 4, panel B), suggesting that further optimization of PCRs might also allow complete coverage for these samples. Furthermore, even incomplete genome sequences can provide valuable information during an outbreak, allowing analysis of individual genes and the tracing of transmission chains (*12*). 

Using this updated protocol, we achieve a sustained capacity of 4 full-length genomes per day for a single person conducting the laboratory work using 2 MinION devices (Figure 1, panels A, B). However, with the exception of the first 2 sequencing runs, bioinformatics analysis during this mission was mainly completed after returning to the NIH, to maximize the time for raw data acquisition (online Technical Appendix).

**Figure 2:**
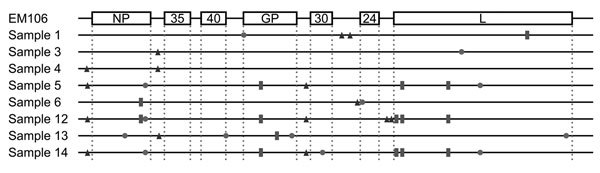
Observed mutations in the 8 fully nanopore-sequenced Ebola-positive blood samples compared to a reference sequence from June 2014 (SLI/Makona-EM106, GenBank accession number KM233036.1). Squares indicate nonsynonymous mutations, circles indicate synonymous changes, and triangles indicate changes in noncoding regions.

Phylogenetic analysis of the complete genomes generated in Monrovia, Liberia, showed them being clearly distinct from Sierra Leone or early Guinea sequences of EBOV-Makona (Technical Appendix Appendix 5) but clustering well with all other sequences found in samples from Liberia. These results suggest that EBOV in Liberia resulted from a single introduction or a limited number of introductions with genetically similar viruses. When analyzing the obtained full-length sequences and comparing them to a consensus sequence from the outbreak (*13*), we observed few mutations, most in noncoding regions or synonymous mutations (Figure 2); none affected siRNA target sequences or the diagnostic targets used in the CDC/NIH laboratory. 

Using Bayesian analysis including these sequences, we estimated the nucleotide substitution rate during the outbreak at 1.36 × 10^−3^, consistent with recently published values (*5*,*13*–*15*). In a root-to-tip-analysis, the sequences we obtained showed substitution rates comparable to other sequences from the outbreak (online Technical Appendix Figure 6). Overall, these data suggest that EBOV has remained relatively stable genetically during the outbreak.

## Conclusions

We found that, because of the device’s small size and comparatively modest resource requirements, nanopore sequencing has tremendous potential for use in remote and resource-limited areas, and its implementation could revolutionize the capacity of public health professionals to perform sequencing during future disease outbreaks. Although we used a directed approach to sequencing, approaches not dependent on prior pathogen identification (i.e. for diagnostic use of the MinION device) are currently being developed (*7*) and will even further increase this technology’s usefulness in future outbreaks.

**Technical Appendix.** Laboratory procedures, phylogenetic analysis of Ebola virus cDNA genomes, and bioinformatics scripts used in study of nanopore sequencing as a rapidly deployable Ebola outbreak tool.
