# Supplementary material for: Nanopore Sequencing as a Rapidly Deployable Ebola Outbreak Tool
Source: Emerg Infect Dis. 2016 Feb;22(2):331–4. doi: 10.3201/eid2202.151796 (PMC4734547; doi:10.3201/eid2202.151796)
Supplement: Supplementary file 1 — Technical Appendix. Laboratory procedures, phylogenetic analysis of Ebola virus cDNA genomes, and bioinformatics scripts used in study of nanopore sequencing as a rapidly deployable Ebola outbreak tool. [file 15-1796-Techapp-s1.pdf]

Article DOI: http://dx.doi.org/10.3201/eid2202.151796

Nanopore Sequencing as a Rapidly Deployable Ebola Outbreak Tool

Technical Appendix

Detailed Materials and Methods

Blood and Virus Samples

Deidentified frozen blood samples or viral transport medium (VTM; from oral swabs) from deceased patients that had tested positive for Ebola virus between November 2014 and January 2015 were thawed and 140 µL of blood or VTM was inactivated in a portable glovebox at the diagnostic laboratory on the ELWA campus in Liberia, Monrovia. RNA was then extracted by using the QIAamp viral RNA mini kit (QIAGEN, Valencia, CA, USA) following the manufacturer's instructions, but with 2 additional AW1 wash steps.

RT-PCRs and Cleanup

To avoid any potential impact on the diagnostic services, all work related to sequencing was performed in a physically separate dedicated area by personnel not involved in diagnostics. Two-step RT-PCRs were performed as previously described (*1*,*2*), but using 5 µL of RNA instead of 1 µL of RNA as starting material. For the RT primer this sequence was used: CGGACACACAAAAAGAAAGAAG. For the second PCRs, 0.8 µL of the PCR product from the first PCR was used as input material without further purification (each PCR product of the first PCR step served as template for 2-second PCR reactions). Primer pairs and cycling conditions are provided in Technical Appendix Tables 1 and 2. After the second PCR, DNA was purified by using Agencourt AMPure XP PCR purification beads (Beckman Coulter, Krefeld, Germany). All 24 RT-PCR products from 1 sample were purified in a total of 2 purification reactions: 40 µL of RT-PCR product from each of the 12 reactions was pooled, and 720 µL of Agencourt beads was added. Samples were incubated for 5 minutes and then placed on a magnetic Agencourt SPRIStand (Beckman Coulter) for 5 minutes. The supernatant was carefully aspirated off the pellet, leaving 10–20 µL of supernatant on the pellet. Then 1.2 mL of 70% ethanol was added to the samples without disturbing the pellet, and they were incubated on the

magnet for 30 seconds. The supernatant was then aspirated completely, and a second wash was performed with 800 µL of ethanol. After the second wash step, the tubes were briefly (≈3 seconds) spun down in a tabletop centrifuge, placed back onto the magnet, and any additional supernatant was removed by using a P10 pipette. The pellets were air-dried on the magnet for ≈2 minutes, before being removed from the magnet and carefully resuspended in 60 µL of EB buffer (QIAGEN). Samples were incubated for 10 minutes, then they were placed back onto the magnet, incubated for 1 minute to allow beads to pellet, and 50 µL of supernatant was carefully removed. The eluates from the 2 purifications (corresponding to 1 patient sample) were then pooled and used for library preparation and sequencing. For visualization, 5 µL of pooled PCR products were run on a 1% agarose gel, stained for 3 minutes in 100× FastBlast (BioRad), rinsed and destained in water, and documented by using an IPhone 4 with a white laptop screen serving as a light box.

MinION Runs

Library preparation was done by using the Genomic DNA Sequencing Kit SQK-MAP004 (Oxford Nanopore Technologies, Oxford, UK [ONT]) following the manufacturer's instructions. Samples were analyzed on a MinION sequencing device using R7.3 FlowCells (ONT) connected to a laptop running the MinKNOW software 0.48.2.12 (ONT). Internet connectivity was provided through a cellular network (Novafone Inc., Monrovia, Liberia) by using a wireless 4G router. Base calling was done by using the ONT Metrichor software version 2.25.1. Due to restrictions in personnel (i.e., the fact that most of the sequencing work in Liberia was done by a single person) and the need to perform the base calling by using a cloud-server, which required upload of the primary data via a 4G cellular network in Liberia, the bioinformatics aspect of the work was done on-site only for the first 2 sequencing runs. After we had demonstrated that this is in principle feasible, we decided to save the remaining raw data temporarily on a portable hard drive, and base calling and the subsequent bioinformatics analysis was done after return to the Rocky Mountain Laboratories, to maximize the generation of raw data. Base calling algorithms that can be run locally, without the need for an internet connection, are currently being developed, and by including a person dedicated to the bioinformatics work on future outbreak missions it should be possible to do this aspect of workflow rapidly on-site, with the same capacity than the laboratory work.

Consensus Calling

All consensus calling was done by using an Ubuntu 14.04 linux environment running under Oracle VM VirtualBox 4.3.20 (Oracle.com). FASTA sequences were extracted from the fast5 files returned by Metrichor by using Poretools 0.5.1 (*3*), and were aligned to a consensus of sequences previously observed in the West African outbreak (*2*) by using lastal 393 (*4*). In this alignment, sequences corresponding to primer sequences incorporated in the PCR product were identified and cropped by using last2fasta.pl (see Bioinformatics Scripts section), and the resulting FASTA file with cropped sequences was realigned by using lastal. The resulting alignment was converted into a SAM file and a pileup was constructed by using Samtools 0.1.19 (*5*). Nucleotide counts for each position were extracted from the pileup by using pileup2nucl.pl, and the consensus sequence was identified by using callnucl.pl.

Calculation of a Theoretical Probability for a Miscalled Base

To estimate the effect of read-depth on the overall reliability of the data, a theoretical probability for a miscalled base (TPMB) was calculated. This value was based on the observation that in our hands plasmid DNA with a known sequence could be sequenced with an accuracy of 84.13%. For a single nucleotide position the TPMB was then calculated for a read-depth from 1 to 170 as the sum of probabilities for at least 50% miscalls using the binomial probability formula (results of these calculations are shown in Technical Appendix Figure 1 panel E). For read depths greater than 170, the TPMB was approximated using regression analysis of these data, as shown in Technical Appendix Figure 1, panel E. For calculating the TPMB across a whole EBOV genome, TPMBs of 2 neighboring nucleotides t_1 and t_2 were combined as $t_{1,2} = (t_1 \times [1-t_2]) + ([1-t_1] \times t_2) + (t_1 \times t_2)$, to give the probability that at $\geq$1 base in the dinucleotide is being miscalled. Then, TPMBs of 2 neighboring dinucleotides were combined in the same fashion, and this process was continued for increasingly larger fragments of the genome, until TPMBs of all nucleotides across the whole length of the genome had been considered. Factors that were not taken into consideration in this estimation were errors introduced by the PCR-amplification steps, or the possibility of a non-random distribution of errors. However, it has to be noted that no obvious non-random distribution of errors in our sequencing data was observed.

Phylogenetic Analysis

For Bayesian coalescent phylogeny, 296 Ebola virus cDNA genomes were aligned by using ClustalX2 (*6*) and this alignment was inspected and manually improved. Sample collection

dates were added to the sequence identifiers to allow serial coalescent analysis. This multiple sequence alignment was input into BEAST v1.8.2 (*7*) to calculate a Bayesian coalescent phylogeny. For this analysis we used the HKY substitution model (*8*), a lognormal relaxed uncorrelated clock model (*9*), and the Bayesian skygrid tree model (*10*). For the clock model the CTMC rate reference prior (*11*) was specified. Four independent 40 million generation runs were performed, of which 3 ran to completion without fatal errors. The 3 successful runs converged to roughly identical parameter estimates so the run with the highest ESS values was used to estimate the phylogeny. To estimate the phylogeny the first 10% of the MCMC samples were discarded as burn-in and a maximum clade credibility tree was derived from the remaining 9001 trees.

For Root-To-Tip analysis, Bayesian analysis was performed by using MrBayes 3.2.5 (http://mrbayes.sourceforge.net/), with a with general time-reversable substitution model with a percent of site invariant and gamma-distributed rate heterogeneity across sites. The analysis was terminated after 2,028,000 generations, as it was determined that it had converged. Root-to-tip distances were calculated by using TreeStat v1.8.2 (http://tree.bio.ed.ac.uk/software/treestat/), and the scatter plot and linear regression were performed in R v3.2.2.

References

<jrn>1. Hoenen T, Groseth A, Feldmann F, Marzi A, Ebihara H, Kobinger G, et al. Complete genome sequences of three ebola virus isolates from the 2014 outbreak in west Africa. Genome Announc. 2014;2:e01331–14. **PMID: 25523781** http://dx.doi.org/10.1128/genomeA.0331-14</jrn>

<jrn>2. Hoenen T, Safronetz D, Groseth A, Wollenberg KR, Koita OA, Diarra B, et al. Virology. Mutation rate and genotype variation of Ebola virus from Mali case sequences. Science. 2015;348:117–9.**PMID: 25814067** http://dx.doi.org/10.1126/science.aaa5646</jrn>

<jrn>3. Loman NJ, Quinlan AR. Poretools: a toolkit for analyzing nanopore sequence data. Bioinformatics. 2014;30:3399–401.**PMID: 25143291** http://dx.doi.org/10.1093/bioinformatics/btu555</jrn>

<jrn>4. Kielbasa SM, Wan R, Sato K, Horton P, Frith MC. Adaptive seeds tame genomic sequence comparison. Genome Res. 2011;21:487–93.**PMID: 21209072** http://dx.doi.org/10.1101/gr.113985.110</jrn>

<jrn>5. Li H, Handsaker B, Wysoker A, Fennell T, Ruan J, Homer N, et al. The Sequence Alignment/Map format and SAMtools. Bioinformatics. 2009;25:2078–9.**PMID: 19505943** http://dx.doi.org/10.1093/bioinformatics/btp352</jrn>

<jrn>6. Larkin MA, Blackshields G, Brown NP, Chenna R, McGettigan PA, McWilliam H, et al. Clustal W and Clustal X version 2.0. Bioinformatics. 2007;23:2947–8. **PMID: 17846036** http://dx.doi.org/10.1093/bioinformatics/btm404</jrn>

<jrn>7. Drummond AJ, Suchard MA, Xie D, Rambaut A. Bayesian phylogenetics with BEAUti and the BEAST 1.7. Mol Biol Evol. 2012;29:1969–73. **PMID: 22367748** http://dx.doi.org/10.1093/molbev/mss075</jrn>

<jrn>8. Hasegawa M, Kishino H, Yano T. Dating of the human-ape splitting by a molecular clock of mitochondrial DNA. J Mol Evol. 1985;22:160–74. **PMID: 3934395** </jrn>

<jrn>9. Drummond AJ, Ho SY, Phillips MJ, Rambaut A. Relaxed phylogenetics and dating with confidence. PLoS Biol. 2006;4:e88. **PMID: 16683862** http://dx.doi.org/10.1371/journal.pbio.0040088</jrn>

<jrn>10. Gill MS, Lemey P, Faria NR, Rambaut A, Shapiro B, Suchard MA. Improving Bayesian population dynamics inference: a coalescent-based model for multiple loci. Mol Biol Evol. 2013;30:713–24. **PMID: 23180580** http://dx.doi.org/10.1093/molbev/mss265</jrn>

<jrn>11. Ferreira MAR, Suchard MA. Bayesian analysis of elapsed times in continuous-time Markov chains. Can J Stat. 2008;36:355–68. http://dx.doi.org/10.1002/cjs.5550360302</jrn>

Technical Appendix Table 1. Primer sequences

First PCR	Second PCR	
fwd: GAGTGCGGACAGTTTCCTTC<br>rev: GATGAATGCTGATGACACACTG	rxn 1	fwd: GGCCAAGCATGGAGAGTATG<br>rev: CAAGCTCGGGGAATGTCAC
	rxn 2	fwd: GGGTGGACAACAGAAAAACAG<br>rev: CAAGCTCGGGGAATGTCAC
fwd: CGAAGCCAAACCCGAAGATG<br>rev: GAGAGCATCTTGCATTGTGTAC	rxn 1	fwd: CCCCTCAATGTGCCCTAATTC<br>rev: GTCGCCTCACAATATCCTTCTAG
	rxn 2	fwd: GCGTAATCTTCATCTCTCTTAG<br>rev: CCATCCTGTCCACCAATTGTC
fwd: CTTGACATCTCTGAGGCAAC<br>rev: GGGTGTGATTTACAGCTAAATGC	rxn 1	fwd: CGAACCACATGATTGGACCAAG<br>rev: CTCATCAGACCTCCGCATTAATC
	rxn 2	fwd: ATATGAGAGAGGACGCCCCC<br>rev: CAGTGAGGATTTATCTGTGGTTAAAC
fwd: GTTAACTTGACATCTCTGCCTTC<br>rev: TGTCGTGAGGATGTACATGATC	rxn 1	fwd: CAAGGCACTATCAGGCAATG<br>rev: CTGCATCAGTCTCTAAGGG
	rxn 2	fwd: CCTCACAAATTCAGTACCAAACG<br>rev: CCGATAGTCCAGCTTATTCG
fwd: CAACCTGGTGGGAAACCATTC<br>rev: TGGACACACAAAAAAGAAGAAATAG	rxn 1	fwd: CCGAGAAAATGAATTGATTTATGAC<br>rev: AGTTAAATGACTTAGCCAGTATGG
	rxn 2	fwd: CCTCACAAATTCAGTACCAAACG<br>rev: TGGACACACAAAAAAGAAGAAATAG
fwd: CGGACACACAAAAAGAAAGAAG<br>rev: CATGGTGAGGTCTCCTGGAG	rxn 1	fwd: CGGACACACAAAAAGAAAGAAG<br>rev: CCTGTTTGCGTTCCTTGACTAT
	rxn 2	fwd: CATGGCAATCCTGCAACATC<br>rev: GATGAATGCTGATGACACACTG
fwd: CCCCTCAATGTGCCCTAATTC<br>rev: CACACGGTAACTGGAGAGC	rxn 1	fwd: GAGACACTCCATCGAATCCAC<br>rev: GGGTCCTCCGTTGCATTGAC
	rxn 2	fwd: GCGTAATCTTCATCTCTCTTAG<br>rev: CTCGATAATTCTCTCTGGATGATG
fwd: ATATGAGAGAGGACGCCCCC<br>rev: GGGTGTGATTTACAGCTAAATGC	rxn 1	fwd: ATATGAGAGAGGACGCCCCC<br>rev: ATGCAGGGGCAAAGTCATTAG
	rxn 2	fwd: GGTGGAAAGTTTATTGGGCTG<br>rev: GGGTGTGATTTACAGCTAAATGC

First PCR	Second PCR	
fwd: TCTCGAACCATTGTGCTTGG<br>rev: TGTCGTGAGGATGTACATGATC	rxn 1	fwd: CCTCACAAATTCAGTACCAAACG<br>rev: CCGATAGTCCAGCTTATTCG
	rxn 2	fwd: CTGGACAAGTATTTCATGTGCTC<br>rev: CAGCTGTTTGCCTTGGAAAAATG
fwd: CGAGAAAATGAATTGATTTATGAC<br>rev: TGGACACACAAAAAAGAAGAAATAG	rxn 1	fwd: GAGATCCGTCATTGATACCACAG<br>rev: TGGACACACAAAAAAGAAGAAATAG
	rxn 2	fwd: ATGCCACACCAAAACCATCTC<br>rev: TGGACACACAAAAAAGAAGAAATAG
fwd: CTGGACAAGTATTTCATGTGCTC<br>rev: AGTTAAATGACTTAGCCAGTATGG	rxn 1	fwd: CTCCGAATGATTGAGATGGATG<br>rev: TGTCGTGAGGATGTACATGATC
	rxn 2	fwd: CAACCTGGTGGGAAACCATTC<br>rev: GCCGACTTAAAATTCTCTATTTCC
fwd: CAAGGCACTATCAGGCAATG<br>rev: CTGCATCAGTCTCTAAGGG	rxn 1	fwd: CAGTTTTGAAGCTGCACTATG<br>rev: GGGTGTGATTTACAGCTAAATGC
	rxn 2	fwd: CAGTTTTGAAGCTGCACTATG<br>rev: GATATTGTGGTAGTAGATACTCGAG

Technical Appendix Table 2. Cycling conditions

First PCR			Nested PCR		
	30 min	98°C		30” min	98°C
10 ×	15”<br>30”<br>90”	98°C<br>59 –54.5°C (–0.5°C / cycle)<br>72°C	10 ×	15”<br>30”<br>60”	98°C<br>59 –54.5°C (–0.5°C / cycle)<br>72°C
30 ×	15”<br>30”<br>90”	98°C<br>54°C<br>72°C	30 ×	15”<br>30”<br>60”	98°C<br>54°C<br>72°C
	3′	72°C		3′	72°C

Technical Appendix Figure 1. Initial testing of MinION sequencing. A) Read depth plot for Ebola virus Makona. A blood sample from a non-human primate infected with Ebola virus Makona was subjected to the procedure shown in Figure 1, panel A. The read depth for each position in the genome on a log10-scale is shown. B) Comparison of a Sanger chromatogram and MinION read data. A peak intensity chromatogram from Sanger-sequencing and the corresponding number of reads displaying each nucleotide from a MinION sequencing run of the same random 30 nt region (nt 400 to 430) of the sample in panel A are shown. C) Read accuracy. Plasmid DNA with a known sequence was sequenced using the MinION device. The percentage of positions with a given accuracy (% of correct calls) is shown in 2% intervals on a linear scale. D) Frequency of second-most called nucleotide (N2). The percentage of positions with a given frequency for the second-most called nucleotide, compared to the dominant nucelotide, is shown in 1% intervals on a log scale. E) Probability for a miscall as a function of read depth. The probability that at least half of the reads correspond to an incorrect nucleotide for a given read depth is shown. In black a regression curve for read depths between 70 and 170 is shown, together with the corresponding formula which was used to approximate the error probability for large read depths (>170). F) Theoretical probability for a miscalled base as a function of read depth. The probability for at $\geq$1 miscalled nucleotide in a complete genome is shown as a function of the read depth.

Technical Appendix Figure 2. Effect of an external heat sink on MinION temperature. A) Improvised external heat sink. The heat sink consisted of a ≈30 × 30 cm metal plate, onto which the sequencing devices were placed. B) Device temperatures with and without external heat sink. Temperature data recorded by the MinION sequencing device from 2 representative 12 hour runs are shown, 1 with the

device sitting on the external heat sink, and 1 on a plastic table, but under otherwise identical conditions (e.g., time of day, external temperature).

Technical Appendix Figure 3. Initial MinION results and optimization of workflow. A) Read depth plot of an initial MinION run. A blood sample was sequenced under field conditions by using the workflow established under laboratory conditions. The read depth for each position in the genome is shown on a log10 scale. B) Optimization of PCRs. Three blood samples were subjected to RT-PCR as outlined in the workflow depicted in Technical Appendix Figure 1, panel A, by using 1 μL RNA as starting material for single PCRs, 5 μL RNA as material for single PCRs, or 5 μL RNA as starting material for nested PCRs. PCR products from each sample were pooled and purified, and 5 μL of the purified products was visualized by gel electrophoresis.

Technical Appendix Figure 4. (A) and (B) Read depth plots of representative samples. The read depths for each position in the genomes of a high (panel A, sample 13) and a low (panel B, sample 9) virus load sample on a log10-scale are shown.

Technical Appendix Figure 5. Phylogenetic analysis of determined full-genome sequences. A Bayesian tree of 296 sequences from the West African outbreak is shown. Branch colors indicate posterior probability as shown in the legend, with terminal branches being shown in black. The x-axis indicates time in years before acquisition of the last sample (March 12th, 2015). Origin countries of the samples are indicated. The blow-out shows sequences from Liberia and Mali. Bold text indicates sequences determined as a part of this study.

Technical Appendix Figure 6. A root-to-tip analysis was performed using for the sequences analyzed in Figure 2. The Liberian sequences obtained by us are highlighted in red, and the blue line shows a linear regression curve of all samples, with the inferred rate of substitution from this regression curve indicated.

Bioinformatics Scripts

Bash script for bioinformatics workflow

```
#!/bin/bash
if [ "$#" -ne 2 ]; then
echo
echo "usage: MinION_CGen <reference_file_without_ending> <dir>"
echo
exit 0
fi
# REFFILE contains reference file name without fasta, SEQDIR contains
directory with files
REFFILE=$1
SEQDIR=$2
echo "extracting all reads"
poretools fasta $SEQDIR > sequences.fasta
echo "generating alignment"
lastdb -Q 0 $REFFILE.index $REFFILE.fasta
```

```
lastal -s 2 -T 0 -Q 0 -a 1 $REFFILE.index sequences.fasta | last-map-probs >
sequences.last
echo "cropping primer fragments from sequences"
cat sequences.last | last2fasta_v4.pl >cropped.fasta
echo "realigning cropped sequences"
lastal -s 2 -T 0 -Q 0 -a 1 $REFFILE.index cropped.fasta | last-map-probs >
cropped.last
echo "generating sorted SAM file"
maf-convert sam cropped.last > cropped.sam
samtools view -T $REFFILE.fasta -bS cropped.sam | samtools sort -
cropped.last.sorted
samtools index cropped.last.sorted.bam
echo "generating pileup"
samtools mpileup -BQ 0 -d 1000000 -f $REFFILE.fasta cropped.last.sorted.bam
>pileup
echo "calling consensus"
cat pileup | pileup2nucl.pl >nucl
cat nucl | callnucl.pl >consensus
```

last2fasta_v4.pl:

```
#!/usr/bin/perl
use warnings;
use strict;
# numbers indicate first and last nucleotide in primer
my @startRanges = ([673, 692],
[1330, 1349],
[1981, 2001],
[2662, 2681],
[3313, 3332],
[3943, 3963],
[4609, 4629],
[5291, 5311],
[5923, 5944],
[6570, 6590],
[7223, 7242],
[7867, 7888],
[8520, 8539],
[9168, 9188],
[9780, 9802],
[10454, 10474],
[11150, 11172],
[11823, 11842],
[12471, 12490],
[13102, 13124],
[13725, 13747],
[14398, 14419],
[15052, 15072],
[15702, 15726],
[16324, 16345],
[16962, 16984],
[17620, 17640],
[18233, 18257]);
my @stopRanges = ([747, 766],
[1440, 1458],
[2057, 2076],
[2739, 2760],
[3394, 3414],
[4030, 4048],
[4679, 4700],
```

```
[5374, 5393],
[6010, 6032],
[6645, 6664],
[7294, 7312],
[7961, 7981],
[8594, 8617],
[9244, 9262],
[9891, 9913],
[10529, 10549],
[11226, 11251],
[11895, 11916],
[12549, 12571],
[13192, 13216],
[13837, 13855],
[14492, 14512],
[15124, 15143],
[15776, 15798],
[16426, 16447],
[17046, 17069],
[17692, 17715],
[18367, 18388]);
my $line;
my $lineIdx = 0;
my $template;
my $read;
# store next relevant line into $line
sub nextLine
{
while (1)
{
$line = <>;
exit unless defined $line; # end of file
$lineIdx++;
next if $line =~ m/^\s*(#.*)?$/; # blank or comment line
chomp $line;
return;
}
}
# remove insertions from reads
sub remove_insertions
{
my $inspos = index($template,"-");
while ($inspos > -1)
{
$template = substr($template,0,$inspos) . substr($template,$inspos+1);
$read = substr($read,0,$inspos) . substr($read,$inspos+1);
$inspos = index($template,"-");
}
}
while (1)
{
# first line
&nextLine;
unless ($line =~ m/^a\s/)
{
die "unexpected line $lineIdx (expected 'a ...'): $line\n";
}
# second line
```

```
&nextLine;
unless ($line =~ m/^s\s+\S+\s+(\d+)\s+(\d+)(?:\s+\S+){2}\s+([ACGT\-]+)/)
{
die(”unexpected line $lineIdx (expected 's ... Start Length ...'):”
. “$line\n”);
}
my $start = $1;
my $stop = $start + $2;
$template = $3;
$start++;
# third line
&nextLine;
unless ($line =~ m/^s\s+(\S+)(?:\s+\S+){4}\s+(\S+)/)
{
die(”unexpected line $lineIdx (expected 's Name ... Sequence ...'):”
. “$line\n”);
}
my $name =$1;
$read = $2;
&remove_insertions;
# eventually strip from line end
foreach (@stopRanges)
{
my $first = $_->[0];
my $last = $_->[1];
if ($first <= $stop and $stop <= $last)
{
$read = substr $read, 0, $first - $stop - 1;
last;
}
}
# eventually strip from line beginning
foreach (@startRanges)
{
my $first = $_->[0];
my $last = $_->[1];
if ($first <= $start and $start <= $last)
{
$read = substr $read, $last - $start + 1;
last;
}
}
# filter out hyphens
$read =~ tr/-//d;
# dump fasta record
print “>$name\n”;
print “$read\n”;
}
```

pileup2nucl.pl:

```
#!/usr/bin/perl
use warnings;
use strict;
my $refbase;
my $callstring;
my $lineindex = 0;
my $position;
my %count = (
A => 0,
```

```
C => 0,
G => 0,
T => 0
);
# get next reference base and callstring
sub nextLine
{
my $line;
my @content;
$line = <>;
&exitprogram unless defined $line; # end of file
@content = split(" ", $line);
$position = $content[1];
$refbase = $content[2];
$callstring = $content[4];
$lineindex++;
return;
}
sub exitprogram
{
$lineindex++;
while ($lineindex<18959)
{
print "$lineindex\tA 0\tC 0\tG 0\tT 0\n";
$lineindex++;
}
exit;
}
sub removeInDels
{
my $croppedstring = "";
my $offset;
my $nextindel = index($callstring,"-");
while ($nextindel>0)
{
$callstring =~ /\-(\d+)/;
$offset = $nextindel + $1 + length($1) + 1;
$croppedstring = $croppedstring . substr($callstring,0,$nextindel);
$callstring = substr($callstring,$offset);
$nextindel = index($callstring,"-");
}
$callstring = $croppedstring . $callstring;
$croppedstring = "";
$nextindel = index($callstring,"+");
while ($nextindel>0)
{
$callstring =~ /\+(\d+)/;
$offset = $nextindel + $1 + length($1) + 1;
$croppedstring = $croppedstring . substr($callstring,0,$nextindel);
$callstring = substr($callstring,$offset);
$nextindel = index($callstring,"+");
}
$callstring = $croppedstring . $callstring;
}
while (1)
{
&nextLine;
while ($lineindex<$position)
```

```
{
print "$lineindex\tA 0\tC 0\tG 0\tT 0\n";
$lineindex++;
}
&removeInDels;
$count{'A'} = ($callstring =~ tr/A//);
$count{'C'} = ($callstring =~ tr/C//);
$count{'G'} = ($callstring =~ tr/G//);
$count{'T'} = ($callstring =~ tr/T//);
$count{$refbase} = ($callstring =~ tr/\.//);
print "$lineindex\tA $count{'A'}\tC $count{'C'}\tG $count{'G'}\tT
$count{'T'}\n";
}
```

callnucl.pl:

```
#!/usr/bin/perl
use warnings;
use strict;
use List::Util qw[min max];
my $A;
my $C;
my $G;
my $T;
my $depth;
my $separation=1.0;
# get next reference base and callstring
sub nextLine
{
my $line;
my @content;
$line = <>;
exit unless defined $line; # end of file
@content = split(/\s/, $line);
$A = $content[2];
$C = $content[4];
$G = $content[6];
$T = $content[8];
return;
}
MAIN: while (1)
{
&nextLine;
$depth=$A+$C+$G+$T;
if ($depth==0)
{
print "n";
next MAIN;
}
if ($A >= max($C,$G,$T))
{
if ($A > (max($C,$G,$T)*$separation))
{
print "a";
next MAIN;
}
if ($C > (max($G,$T)*$separation))
{
print "m";
next MAIN;
```

```
}
if ($G > (max($C,$T)*$separation))
{
print "r";
next MAIN;
}
if ($T > (max($C,$G)*$separation))
{
print "w";
next MAIN;
}
}
if ($C >= max($A,$G,$T))
{
if ($C > (max($A,$G,$T)*$separation))
{
print "c";
next MAIN;
}
if ($A > (max($G,$T)*$separation))
{
print "m";
next MAIN;
}
if ($G > (max($A,$T)*$separation))
{
print "s";
next MAIN;
}
if ($T > (max($A,$G)*$separation))
{
print "y";
next MAIN;
}
}
if ($G >= max($A,$C,$T))
{
if ($G > (max($A,$C,$T)*$separation))
{
print "g";
next MAIN;
}
if ($A > (max($C,$T)*$separation))
{
print "r";
next MAIN;
}
if ($C > (max($A,$T)*$separation))
{
print "s";
next MAIN;
}
if ($T > (max($A,$C)*$separation))
{
print "k";
next MAIN;
}
}
```

```
if ($T >= max($A,$C,$G))
{
if ($T > (max($A,$C,$G)*$separation))
{
print "t";
next MAIN;
}
if ($A > (max($C,$G)*$separation))
{
print "w";
next MAIN;
}
if ($C > (max($A,$G)*$separation))
{
print "y";
next MAIN;
}
if ($G > (max($A,$C)*$separation))
{
print "k";
next MAIN;
}
}
if (($A*$separation) <= min($C,$G,$T))
{
print "b";
next MAIN;
}
if (($C*$separation) <= min($A,$G,$T))
{
print "d";
next MAIN;
}
if (($G*$separation) <= min($A,$C,$T))
{
print "h";
next MAIN;
}
if (($T*$separation) <= min($A,$C,$G))
{
print "v";
next MAIN;
}
print "n";
}
```
